# Validating module network learning algorithms using simulated data

**DOI:** 10.1186/1471-2105-8-S2-S5

**Published:** 2007-05-03

**Authors:** Tom Michoel, Steven Maere, Eric Bonnet, Anagha Joshi, Yvan Saeys, Tim Van den Bulcke, Koenraad Van Leemput, Piet van Remortel, Martin Kuiper, Kathleen Marchal, Yves Van de Peer

**Affiliations:** 1 Bioinformatics & Evolutionary Genomics, Department of Plant Systems Biology, VIB/Ghent University, Technologiepark 927, B-9052 Ghent, Belgium; 2 ESAT-SCD, K.U.Leuven, Kasteelpark Arenberg 10, B-3001 Leuven, Belgium; 3 ISLab, Department of Mathematics and Computer Science, University of Antwerp, Middelheimlaan 1, B-2020 Antwerpen, Belgium; 4 CMPG, Department Microbial and Molecular Systems, K.U.Leuven, Kasteelpark Arenberg 20, B-3001 Leuven, Belgium

## Abstract

**Background:**

In recent years, several authors have used probabilistic graphical models to learn expression modules and their regulatory programs from gene expression data. Despite the demonstrated success of such algorithms in uncovering biologically relevant regulatory relations, further developments in the area are hampered by a lack of tools to compare the performance of alternative module network learning strategies. Here, we demonstrate the use of the synthetic data generator SynTReN for the purpose of testing and comparing module network learning algorithms. We introduce a software package for learning module networks, called LeMoNe, which incorporates a novel strategy for learning regulatory programs. Novelties include the use of a bottom-up Bayesian hierarchical clustering to construct the regulatory programs, and the use of a conditional entropy measure to assign regulators to the regulation program nodes. Using SynTReN data, we test the performance of LeMoNe in a completely controlled situation and assess the effect of the methodological changes we made with respect to an existing software package, namely Genomica. Additionally, we assess the effect of various parameters, such as the size of the data set and the amount of noise, on the inference performance.

**Results:**

Overall, application of Genomica and LeMoNe to simulated data sets gave comparable results. However, LeMoNe offers some advantages, one of them being that the learning process is considerably faster for larger data sets. Additionally, we show that the location of the regulators in the LeMoNe regulation programs and their conditional entropy may be used to prioritize regulators for functional validation, and that the combination of the bottom-up clustering strategy with the conditional entropy-based assignment of regulators improves the handling of missing or hidden regulators.

**Conclusion:**

We show that data simulators such as SynTReN are very well suited for the purpose of developing, testing and improving module network algorithms. We used SynTReN data to develop and test an alternative module network learning strategy, which is incorporated in the software package LeMoNe, and we provide evidence that this alternative strategy has several advantages with respect to existing methods.

## Background

For the past 45 years, research in molecular biology has been based predominantly on reductionist thinking, trying to unravel the complex workings of living organisms by investigating genes or proteins one at a time. In recent years, molecular biologists have come to view the cell from a different, more global perspective. With the advent of fully sequenced genomes and high-throughput functional genomics technologies, it has become possible to monitor molecular properties such as gene expression levels or protein-DNA interactions across thousands of genes simultaneously. As a consequence, it has become feasible to study genes, proteins and their interactions in the context of biological systems rather than in isolation. This novel paradigm has been named 'systems biology' [1].

One of the goals of the systems approach to molecular biology is to reverse engineer the regulatory networks underlying cell function. Particularly transcriptional regulatory networks have received a lot of attention, mainly because of the availability of large amounts of relevant experimental data. Several studies use expression data, promoter motif data, chromatin immunoprecipitation (ChIP) data and/or prior functional information (e.g. GO classifications [2] or known regulatory network structures) in conjunction to elucidate transcriptional regulatory networks [3-17]. Most of these methods try to unravel the *control logic *underlying specific expression patterns. This type of analysis typically requires elaborate computational frameworks. In particular probabilistic graphical models are considered a natural mathematical framework for inferring regulatory networks [8]. Probabilistic graphical models, the best-known representatives being Bayesian networks, represent the system under study in terms of conditional probability distributions describing the observations for each of the variables (genes) as a function of a limited number of parent variables (regulators), thereby reconstructing the regulatory network underlying the observations. Friedman *et al*. pioneered the use of Bayesian networks to learn regulatory networks from expression data [3,4]. In these early studies, each gene in the resulting Bayesian network is associated with its individual regulation program, i.e., its own set of parents and conditional probability distribution. A key limitation of this approach is that a vast number of structural features and distribution parameters need to be learned given only a limited number of expression profiles. In other words, the problem of finding back the real network structure is typically heavily underdetermined. An attractive way to remedy this issue is to take advantage of the inherent modularity of biological networks [18], specifically the fact that groups of genes acting in concert are often regulated by the same regulators. Segal *et al*. [6,19] first exploited this idea by proposing *module networks *as a mathematical model for regulatory networks. Module networks are probabilistic graphical models in which groups of genes, called *modules*, share the same parents and conditional distributions. As the number of parameters to be estimated in a module network is much smaller than in a full Bayesian network, the currently available gene expression data sets can be large enough for the purpose of learning module networks [6,11,12,19].

Despite the demonstrated success of module network learning algorithms in finding biologically relevant regulatory relations [6,11,12,19], there is only limited information about the actual recall and precision of such algorithms [12] and how these performance measures are influenced by the use of alternative module network learning strategies. Having the means to answer the latter question is key to the further development and improvement of the module networks formalism.

The purpose of the present study is twofold. First, we introduce a novel software package for learning module networks, called LeMoNe, which is based on the general methodology outlined in Segal *et al*. [6] but incorporates an alternative strategy for inferring regulation programs.

Second, we demonstrate the use of SynTReN [20], a data simulator that creates synthetic regulatory networks and produces simulated gene expression data, for the purpose of testing and comparing module network learning algorithms. We use SynTReN data to assess the performance of LeMoNe and to compare the behavior of alternative module network learning strategies. Additionally, we assess the effect of various parameters, such as the size of the data set and the amount of noise, on the inference performance. For comparison, we also use LeMoNe to analyze real expression data for *S. cerevisiae *[21] and investigate to what extent the quality of the module networks learned on real data can be automatically assessed using structured biological information such as GO information and ChIP-chip data [9].

## Methods

### Data sets

We used SynTReN [20] to generate simulated data sets for a gene network with 1000 genes of which 105 act as regulators. The topology of the network is subsampled from an *E. coli *transcriptional network [29] by cluster addition, resulting in a network with 2361 edges. All parameters of SynTReN were set to default values, except number of correlated inputs, which was set to 50%. SynTReN generated expression values ranging from 0 (no expression) to 1 (maximal expression) which we normalized to log_2 _ratio values by picking one of the experiments as the control. Except where indicated otherwise, the list of true regulators was given as the list of potential regulators for LeMoNe and Genomica.

For the tests performed on real data, we used an expression compendium for *S. cerevisiae *containing expression data for 173 different experimental stress conditions [21]. The data were obtained in prenormalized and preprocessed form. We used the mean log_2 _values of the expression ratios (perturbation vs. control).

To assess the quality of the regulatory programs learned from real data, we used data on genome-wide binding and phylogenetically conserved motifs for 102 transcription factors from Harbison *et al*. [9]. For a given transcription factor, only genes that were bound with high confidence (significance level *α *= 0.005) and showed motif conservation in at least one other *Saccharomyces *species (besides *S. cerevisiae*) were considered true targets.

### Module networks

Module networks are a special kind of Bayesian networks and were introduced by Segal *et al*. [6,30]. To each gene *i *we associate a random variable *X*_*i *_which can take continuous values and corresponds to the gene's expression level. The distribution of *X*_*i *_depends on the expression level of a set of parent genes Pa_*i *_chosen from a list of potential regulators. If the network formed by drawing directed edges from parent genes to children genes is acyclic, we can define a joint probability distribution for the expression levels of all genes as a product of conditional distributions,

p(x1,...,xN)=∏i=1Npi(xi|{xj:j∈Pai}).     (1)
 MathType@MTEF@5@5@+=feaafiart1ev1aaatCvAUfKttLearuWrP9MDH5MBPbIqV92AaeXatLxBI9gBaebbnrfifHhDYfgasaacH8akY=wiFfYdH8Gipec8Eeeu0xXdbba9frFj0=OqFfea0dXdd9vqai=hGuQ8kuc9pgc9s8qqaq=dirpe0xb9q8qiLsFr0=vr0=vr0dc8meaabaqaciaacaGaaeqabaqabeGadaaakeaacqWGWbaCcqGGOaakcqWG4baEdaWgaaWcbaGaeGymaedabeaakiabcYcaSiabc6caUiabc6caUiabc6caUiabcYcaSiabdIha4naaBaaaleaacqWGobGtaeqaaOGaeiykaKIaeyypa0ZaaebCaeaacqWGWbaCdaWgaaWcbaGaemyAaKgabeaakiabcIcaOiabdIha4naaBaaaleaacqWGPbqAaeqaaOGaeiiFaWNaei4EaSNaemiEaG3aaSbaaSqaaiabdQgaQbqabaGccqGG6aGocqWGQbGAcqGHiiIZieaacqWFqbaucqWFHbqydaWgaaWcbaacbiGae4xAaKgabeaakiabc2ha9jabcMcaPiabc6caUiaaxMaacaWLjaWaaeWaaeaacqaIXaqmaiaawIcacaGLPaaaaSqaaiabdMgaPjabg2da9iabigdaXaqaaiabd6eaobqdcqGHpis1aaaa@5D1F@

This is the standard Bayesian network formalism.

In a module network we assume that genes are partitioned into different sets called *modules*, such that genes in the same module share the same parameters in the distribution function (1). Hence a module network is defined by a partition of {1,...,*N*} into *K *≪ *N *modules A
 MathType@MTEF@5@5@+=feaafiart1ev1aaatCvAUfKttLearuWrP9MDH5MBPbIqV92AaeXatLxBI9gBamrtHrhAL1wy0L2yHvtyaeHbnfgDOvwBHrxAJfwnaebbnrfifHhDYfgasaacH8akY=wiFfYdH8Gipec8Eeeu0xXdbba9frFj0=OqFfea0dXdd9vqai=hGuQ8kuc9pgc9s8qqaq=dirpe0xb9q8qiLsFr0=vr0=vr0dc8meaabaqaciaacaGaaeqabaWaaeGaeaaakeaaimaacqWFaeFqaaa@3821@_*k *_such that ∪k=1KAk
 MathType@MTEF@5@5@+=feaafiart1ev1aaatCvAUfKttLearuWrP9MDH5MBPbIqV92AaeXatLxBI9gBaebbnrfifHhDYfgasaacH8akY=wiFfYdH8Gipec8Eeeu0xXdbba9frFj0=OqFfea0dXdd9vqai=hGuQ8kuc9pgc9s8qqaq=dirpe0xb9q8qiLsFr0=vr0=vr0dc8meaabaqaciaacaGaaeqabaqabeGadaaakeaacqWIQisvdaqhaaWcbaGaem4AaSMaeyypa0JaeGymaedabaGaem4saSeaamrtHrhAL1wy0L2yHvtyaeHbnfgDOvwBHrxAJfwnaGabaOGae8haXh0aaSbaaSqaaiabdUgaRbqabaaaaa@3F88@ = {1,...,*N*} and A
 MathType@MTEF@5@5@+=feaafiart1ev1aaatCvAUfKttLearuWrP9MDH5MBPbIqV92AaeXatLxBI9gBamrtHrhAL1wy0L2yHvtyaeHbnfgDOvwBHrxAJfwnaebbnrfifHhDYfgasaacH8akY=wiFfYdH8Gipec8Eeeu0xXdbba9frFj0=OqFfea0dXdd9vqai=hGuQ8kuc9pgc9s8qqaq=dirpe0xb9q8qiLsFr0=vr0=vr0dc8meaabaqaciaacaGaaeqabaWaaeGaeaaakeaaimaacqWFaeFqaaa@3821@_*k *_∩ A
 MathType@MTEF@5@5@+=feaafiart1ev1aaatCvAUfKttLearuWrP9MDH5MBPbIqV92AaeXatLxBI9gBamrtHrhAL1wy0L2yHvtyaeHbnfgDOvwBHrxAJfwnaebbnrfifHhDYfgasaacH8akY=wiFfYdH8Gipec8Eeeu0xXdbba9frFj0=OqFfea0dXdd9vqai=hGuQ8kuc9pgc9s8qqaq=dirpe0xb9q8qiLsFr0=vr0=vr0dc8meaabaqaciaacaGaaeqabaWaaeGaeaaakeaaimaacqWFaeFqaaa@3821@_*k' *_= ∅ for *k *≠ *k'*, a collection of parent genes ∏_*k *_for each module *k*, and a joint probability distribution

p(x1,...,xN)=∏k=1K∏i∈Akpk(xi|{xj:j∈∏k}).     (2)
 MathType@MTEF@5@5@+=feaafiart1ev1aaatCvAUfKttLearuWrP9MDH5MBPbIqV92AaeXatLxBI9gBaebbnrfifHhDYfgasaacH8akY=wiFfYdH8Gipec8Eeeu0xXdbba9frFj0=OqFfea0dXdd9vqai=hGuQ8kuc9pgc9s8qqaq=dirpe0xb9q8qiLsFr0=vr0=vr0dc8meaabaqaciaacaGaaeqabaqabeGadaaakeaacqWGWbaCcqGGOaakcqWG4baEdaWgaaWcbaGaeGymaedabeaakiabcYcaSiabc6caUiabc6caUiabc6caUiabcYcaSiabdIha4naaBaaaleaacqWGobGtaeqaaOGaeiykaKIaeyypa0ZaaebCaeaadaqeqbqaaiabdchaWnaaBaaaleaacqWGRbWAaeqaaOGaeiikaGIaemiEaG3aaSbaaSqaaiabdMgaPbqabaGccqGG8baFcqGG7bWEcqWG4baEdaWgaaWcbaGaemOAaOgabeaakiabcQda6iabdQgaQjabgIGiolabg+GivpaaBaaaleaaieGacqWFRbWAaeqaaOGaeiyFa0NaeiykaKIaeiOla4caleaacqWGPbqAcqGHiiIZt0uy0HwzTfgDPnwy1egaryqtHrhAL1wy0L2yHvdaiqaacqGFaeFqdaWgaaadbaGaem4AaSgabeaaaSqab0Gaey4dIunaaSqaaiabdUgaRjabg2da9iabigdaXaqaaiabdUealbqdcqGHpis1aOGaaCzcaiaaxMaadaqadaqaaiabikdaYaGaayjkaiaawMcaaaaa@6E4A@

**Figure 1:**
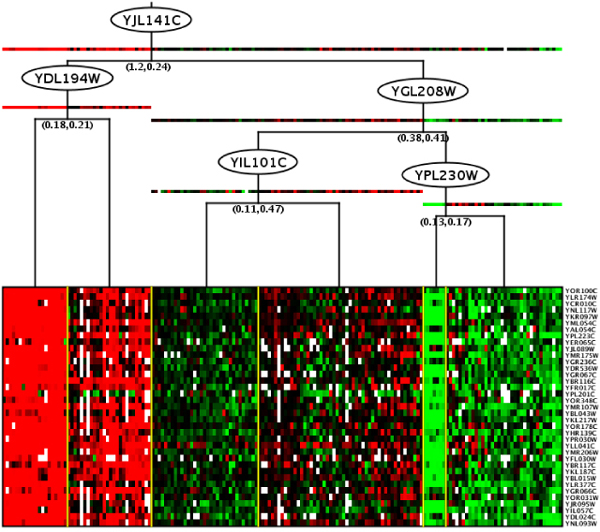
**Sample module learned from the Gasch data set**. Sample module learned from the Gasch data set [21]. Red and green hues indicate upregulation resp. downregulation. The pairs (*x*, *y*) under each split in the regulation tree represent the Bayesian score gain over the split, normalized on the number of genes in the complete network (*x*), and the regulator assignment entropy (*y*).

The conditional distribution *p*_*k *_of the expression level of the genes in module *k *is normal with mean and standard deviation depending on the expression values of the parents of the module through a regression tree that is called the *regulation program *of the module. The tests on the internal nodes of the regression tree are of the form x ≷ s
 MathType@MTEF@5@5@+=feaafiart1ev1aaatCvAUfKttLearuWrP9MDH5MBPbIqV92AaeXatLxBI9gBaebbnrfifHhDYfgasaacH8akY=wiFfYdH8Gipec8Eeeu0xXdbba9frFj0=OqFfea0dXdd9vqai=hGuQ8kuc9pgc9s8qqaq=dirpe0xb9q8qiLsFr0=vr0=vr0dc8meaabaqaciaacaGaaeqabaqabeGadaaakeaacqWG4baEcqqGGaaicqWI9=VBcqqGGaaicqWGZbWCaaa@341D@ for some split value *s*, where *x *is the expression value of the parent associated to the node (Figure 1).

The Bayesian score is obtained by taking the log of the marginal probability of the data likelihood over the parameters of the normal distributions at the leaves of the regression trees with a normal-gamma prior (see [30] and Additional file 1 for more details; the actual expression for the score is in eq. (S5)). Its main property is that it decomposes as a sum of leaf scores of the different modules:

S=∑kSk=∑k∑ℓSk(ℰℓ),     (3)
 MathType@MTEF@5@5@+=feaafiart1ev1aaatCvAUfKttLearuWrP9MDH5MBPbIqV92AaeXatLxBI9gBaebbnrfifHhDYfgasaacH8akY=wiFfYdH8Gipec8Eeeu0xXdbba9frFj0=OqFfea0dXdd9vqai=hGuQ8kuc9pgc9s8qqaq=dirpe0xb9q8qiLsFr0=vr0=vr0dc8meaabaqaciaacaGaaeqabaqabeGadaaakeaaieqacqWFtbWucqGH9aqpdaaeqbqaaiab=nfatnaaBaaaleaacqWGRbWAaeqaaOGaeyypa0daleaacqWGRbWAaeqaniabggHiLdGcdaaeqbqaamaaqafabaGaem4uam1aaSbaaSqaaiabdUgaRbqabaGccqGGOaakt0uy0HwzTfgDPnwy1egaryqtHrhAL1wy0L2yHvdaiqaacqGFWesrdaWgaaWcbaGaeS4eHWgabeaakiabcMcaPaWcbaGaeS4eHWgabeqdcqGHris5aOGaeiilaWIaaCzcaiaaxMaadaqadaqaaiabiodaZaGaayjkaiaawMcaaaWcbaGaem4AaSgabeqdcqGHris5aaaa@526E@

where ℰ
 MathType@MTEF@5@5@+=feaafiart1ev1aaatCvAUfKttLearuWrP9MDH5MBPbIqV92AaeXatLxBI9gBamrtHrhAL1wy0L2yHvtyaeHbnfgDOvwBHrxAJfwnaebbnrfifHhDYfgasaacH8akY=wiFfYdH8Gipec8Eeeu0xXdbba9frFj0=OqFfea0dXdd9vqai=hGuQ8kuc9pgc9s8qqaq=dirpe0xb9q8qiLsFr0=vr0=vr0dc8meaabaqaciaacaGaaeqabaWaaeGaeaaakeaaimaacqWFWesraaa@3785@_ℓ _denotes the experiments that end up at leaf ℓ after traversing the regression tree. A normal-gamma prior ensures that *S*_*k*_(ℰ
 MathType@MTEF@5@5@+=feaafiart1ev1aaatCvAUfKttLearuWrP9MDH5MBPbIqV92AaeXatLxBI9gBamrtHrhAL1wy0L2yHvtyaeHbnfgDOvwBHrxAJfwnaebbnrfifHhDYfgasaacH8akY=wiFfYdH8Gipec8Eeeu0xXdbba9frFj0=OqFfea0dXdd9vqai=hGuQ8kuc9pgc9s8qqaq=dirpe0xb9q8qiLsFr0=vr0=vr0dc8meaabaqaciaacaGaaeqabaWaaeGaeaaakeaaimaacqWFWesraaa@3785@_ℓ_) can be solved explicitly as a function of the sufficient statistics (number of data points, mean and standard deviation) of the leaves of the regression tree (see Additional file 1).

### Learning module regulation programs

For a given assignment of genes to modules, finding a maximum for the Bayesian score (3) consists of finding the optimal partitioning of experiments into 'leaves' ℓ for each module separately, i.e., find a collection of subsets ℰ
 MathType@MTEF@5@5@+=feaafiart1ev1aaatCvAUfKttLearuWrP9MDH5MBPbIqV92AaeXatLxBI9gBamrtHrhAL1wy0L2yHvtyaeHbnfgDOvwBHrxAJfwnaebbnrfifHhDYfgasaacH8akY=wiFfYdH8Gipec8Eeeu0xXdbba9frFj0=OqFfea0dXdd9vqai=hGuQ8kuc9pgc9s8qqaq=dirpe0xb9q8qiLsFr0=vr0=vr0dc8meaabaqaciaacaGaaeqabaWaaeGaeaaakeaaimaacqWFWesraaa@3785@_ℓ _⊂ {1,...,*M*} such that ⋃_ℓ_ℰ
 MathType@MTEF@5@5@+=feaafiart1ev1aaatCvAUfKttLearuWrP9MDH5MBPbIqV92AaeXatLxBI9gBamrtHrhAL1wy0L2yHvtyaeHbnfgDOvwBHrxAJfwnaebbnrfifHhDYfgasaacH8akY=wiFfYdH8Gipec8Eeeu0xXdbba9frFj0=OqFfea0dXdd9vqai=hGuQ8kuc9pgc9s8qqaq=dirpe0xb9q8qiLsFr0=vr0=vr0dc8meaabaqaciaacaGaaeqabaWaaeGaeaaakeaaimaacqWFWesraaa@3785@_ℓ _= {1,...,*M*}, ℰ
 MathType@MTEF@5@5@+=feaafiart1ev1aaatCvAUfKttLearuWrP9MDH5MBPbIqV92AaeXatLxBI9gBamrtHrhAL1wy0L2yHvtyaeHbnfgDOvwBHrxAJfwnaebbnrfifHhDYfgasaacH8akY=wiFfYdH8Gipec8Eeeu0xXdbba9frFj0=OqFfea0dXdd9vqai=hGuQ8kuc9pgc9s8qqaq=dirpe0xb9q8qiLsFr0=vr0=vr0dc8meaabaqaciaacaGaaeqabaWaaeGaeaaakeaaimaacqWFWesraaa@3785@_ℓ _∩ ℰ
 MathType@MTEF@5@5@+=feaafiart1ev1aaatCvAUfKttLearuWrP9MDH5MBPbIqV92AaeXatLxBI9gBamrtHrhAL1wy0L2yHvtyaeHbnfgDOvwBHrxAJfwnaebbnrfifHhDYfgasaacH8akY=wiFfYdH8Gipec8Eeeu0xXdbba9frFj0=OqFfea0dXdd9vqai=hGuQ8kuc9pgc9s8qqaq=dirpe0xb9q8qiLsFr0=vr0=vr0dc8meaabaqaciaacaGaaeqabaWaaeGaeaaakeaaimaacqWFWesraaa@3785@_ℓ _= ∅ for ℓ ≠ ℓ', and

Sk=∑ℓSk(ℰℓ)     (4)
 MathType@MTEF@5@5@+=feaafiart1ev1aaatCvAUfKttLearuWrP9MDH5MBPbIqV92AaeXatLxBI9gBaebbnrfifHhDYfgasaacH8akY=wiFfYdH8Gipec8Eeeu0xXdbba9frFj0=OqFfea0dXdd9vqai=hGuQ8kuc9pgc9s8qqaq=dirpe0xb9q8qiLsFr0=vr0=vr0dc8meaabaqaciaacaGaaeqabaqabeGadaaakeaaieqacqWFtbWudaWgaaWcbaGaem4AaSgabeaakiabg2da9maaqafabaGaem4uam1aaSbaaSqaaiabdUgaRbqabaGccqGGOaakt0uy0HwzTfgDPnwy1egaryqtHrhAL1wy0L2yHvdaiqaacqGFWesrdaWgaaWcbaGaeS4eHWgabeaakiabcMcaPaWcbaGaeS4eHWgabeqdcqGHris5aOGaaCzcaiaaxMaadaqadaqaaiabisda0aGaayjkaiaawMcaaaaa@4851@

is maximal. In particular we do not have to define the parent sets ∏_*k *_of the modules in order to find an optimal partition.

We use a bottom-up hierarchical clustering method to heuristically find a high-scoring partition. At each step of the process we have a collection of binary trees *T_α _*which represent subsets ℰ
 MathType@MTEF@5@5@+=feaafiart1ev1aaatCvAUfKttLearuWrP9MDH5MBPbIqV92AaeXatLxBI9gBamrtHrhAL1wy0L2yHvtyaeHbnfgDOvwBHrxAJfwnaebbnrfifHhDYfgasaacH8akY=wiFfYdH8Gipec8Eeeu0xXdbba9frFj0=OqFfea0dXdd9vqai=hGuQ8kuc9pgc9s8qqaq=dirpe0xb9q8qiLsFr0=vr0=vr0dc8meaabaqaciaacaGaaeqabaWaaeGaeaaakeaaimaacqWFWesraaa@3785@_*α *_of experiments. The binary split of *T_α _*into its children Tα1
 MathType@MTEF@5@5@+=feaafiart1ev1aaatCvAUfKttLearuWrP9MDH5MBPbIqV92AaeXatLxBI9gBaebbnrfifHhDYfgasaacH8akY=wiFfYdH8Gipec8Eeeu0xXdbba9frFj0=OqFfea0dXdd9vqai=hGuQ8kuc9pgc9s8qqaq=dirpe0xb9q8qiLsFr0=vr0=vr0dc8meaabaqaciaacaGaaeqabaqabeGadaaakeaacqWGubavdaWgaaWcbaacciGae8xSde2aaSbaaWqaaiabigdaXaqabaaaleqaaaaa@30D7@ and Tα2
 MathType@MTEF@5@5@+=feaafiart1ev1aaatCvAUfKttLearuWrP9MDH5MBPbIqV92AaeXatLxBI9gBaebbnrfifHhDYfgasaacH8akY=wiFfYdH8Gipec8Eeeu0xXdbba9frFj0=OqFfea0dXdd9vqai=hGuQ8kuc9pgc9s8qqaq=dirpe0xb9q8qiLsFr0=vr0=vr0dc8meaabaqaciaacaGaaeqabaqabeGadaaakeaacqWGubavdaWgaaWcbaacciGae8xSde2aaSbaaWqaaiabikdaYaqabaaaleqaaaaa@30D9@ corresponds to a partition of the set ℰ
 MathType@MTEF@5@5@+=feaafiart1ev1aaatCvAUfKttLearuWrP9MDH5MBPbIqV92AaeXatLxBI9gBamrtHrhAL1wy0L2yHvtyaeHbnfgDOvwBHrxAJfwnaebbnrfifHhDYfgasaacH8akY=wiFfYdH8Gipec8Eeeu0xXdbba9frFj0=OqFfea0dXdd9vqai=hGuQ8kuc9pgc9s8qqaq=dirpe0xb9q8qiLsFr0=vr0=vr0dc8meaabaqaciaacaGaaeqabaWaaeGaeaaakeaaimaacqWFWesraaa@3785@_*α *_into two sets: ℰ
 MathType@MTEF@5@5@+=feaafiart1ev1aaatCvAUfKttLearuWrP9MDH5MBPbIqV92AaeXatLxBI9gBamrtHrhAL1wy0L2yHvtyaeHbnfgDOvwBHrxAJfwnaebbnrfifHhDYfgasaacH8akY=wiFfYdH8Gipec8Eeeu0xXdbba9frFj0=OqFfea0dXdd9vqai=hGuQ8kuc9pgc9s8qqaq=dirpe0xb9q8qiLsFr0=vr0=vr0dc8meaabaqaciaacaGaaeqabaWaaeGaeaaakeaaimaacqWFWesraaa@3785@_*α *_= ℰα1
 MathType@MTEF@5@5@+=feaafiart1ev1aaatCvAUfKttLearuWrP9MDH5MBPbIqV92AaeXatLxBI9gBaebbnrfifHhDYfgasaacH8akY=wiFfYdH8Gipec8Eeeu0xXdbba9frFj0=OqFfea0dXdd9vqai=hGuQ8kuc9pgc9s8qqaq=dirpe0xb9q8qiLsFr0=vr0=vr0dc8meaabaqaciaacaGaaeqabaqabeGadaaakeaat0uy0HwzTfgDPnwy1egaryqtHrhAL1wy0L2yHvdaiqaacqWFWesrdaWgaaWcbaacciGae4xSde2aaSbaaWqaaiabigdaXaqabaaaleqaaaaa@3A7D@ ∪ ℰα2
 MathType@MTEF@5@5@+=feaafiart1ev1aaatCvAUfKttLearuWrP9MDH5MBPbIqV92AaeXatLxBI9gBaebbnrfifHhDYfgasaacH8akY=wiFfYdH8Gipec8Eeeu0xXdbba9frFj0=OqFfea0dXdd9vqai=hGuQ8kuc9pgc9s8qqaq=dirpe0xb9q8qiLsFr0=vr0=vr0dc8meaabaqaciaacaGaaeqabaqabeGadaaakeaat0uy0HwzTfgDPnwy1egaryqtHrhAL1wy0L2yHvdaiqaacqWFWesrdaWgaaWcbaacciGae4xSde2aaSbaaWqaaiabikdaYaqabaaaleqaaaaa@3A7F@. The initial collection consists of trivial trees without children representing single experiments. To proceed from one collection of trees to the next, the pair of trees with highest merge score is merged into a new tree, and the collection of binary trees decreases by one, eventually leading to one hierarchical tree *T*_0 _representing the complete experiment set ℰ
 MathType@MTEF@5@5@+=feaafiart1ev1aaatCvAUfKttLearuWrP9MDH5MBPbIqV92AaeXatLxBI9gBamrtHrhAL1wy0L2yHvtyaeHbnfgDOvwBHrxAJfwnaebbnrfifHhDYfgasaacH8akY=wiFfYdH8Gipec8Eeeu0xXdbba9frFj0=OqFfea0dXdd9vqai=hGuQ8kuc9pgc9s8qqaq=dirpe0xb9q8qiLsFr0=vr0=vr0dc8meaabaqaciaacaGaaeqabaWaaeGaeaaakeaaimaacqWFWesraaa@3785@_0 _= {1,...,*M*}. The simplest merge score is given by the possible gain in Bayesian score by merging two experiment sets:

rα1,α2=Sk(ℰα1∪ℰα2)−Sk(ℰα1)−Sk(ℰα2).     (5)
 MathType@MTEF@5@5@+=feaafiart1ev1aaatCvAUfKttLearuWrP9MDH5MBPbIqV92AaeXatLxBI9gBaebbnrfifHhDYfgasaacH8akY=wiFfYdH8Gipec8Eeeu0xXdbba9frFj0=OqFfea0dXdd9vqai=hGuQ8kuc9pgc9s8qqaq=dirpe0xb9q8qiLsFr0=vr0=vr0dc8meaabaqaciaacaGaaeqabaqabeGadaaakeaacqWGYbGCdaWgaaWcbaacciGae8xSde2aaSbaaWqaaiabigdaXaqabaWccqGGSaalcqWFXoqydaWgaaadbaGaeGOmaidabeaaaSqabaGccqGH9aqpcqWGtbWudaWgaaWcbaGaem4AaSgabeaakiabcIcaOmrtHrhAL1wy0L2yHvtyaeHbnfgDOvwBHrxAJfwnaGabaiab+btifnaaBaaaleaacqWFXoqydaWgaaadbaGaeGymaedabeaaaSqabaGccqGHQicYcqGFWesrdaWgaaWcbaGae8xSde2aaSbaaWqaaiabikdaYaqabaaaleqaaOGaeiykaKIaeyOeI0Iaem4uam1aaSbaaSqaaiabdUgaRbqabaGccqGGOaakcqGFWesrdaWgaaWcbaGae8xSde2aaSbaaWqaaiabigdaXaqabaaaleqaaOGaeiykaKIaeyOeI0Iaem4uam1aaSbaaSqaaiabdUgaRbqabaGccqGGOaakcqGFWesrdaWgaaWcbaGae8xSde2aaSbaaWqaaiabikdaYaqabaaaleqaaOGaeiykaKIaeiOla4IaaCzcaiaaxMaadaqadaqaaiabiwda1aGaayjkaiaawMcaaaaa@6555@

In Additional file 1 we define an alternative merge score related to the Bayesian hierarchical clustering method of [31]. This merge score takes into account the substructure of the trees below Tα1
 MathType@MTEF@5@5@+=feaafiart1ev1aaatCvAUfKttLearuWrP9MDH5MBPbIqV92AaeXatLxBI9gBaebbnrfifHhDYfgasaacH8akY=wiFfYdH8Gipec8Eeeu0xXdbba9frFj0=OqFfea0dXdd9vqai=hGuQ8kuc9pgc9s8qqaq=dirpe0xb9q8qiLsFr0=vr0=vr0dc8meaabaqaciaacaGaaeqabaqabeGadaaakeaacqWGubavdaWgaaWcbaacciGae8xSde2aaSbaaWqaaiabigdaXaqabaaaleqaaaaa@30D7@ and Tα2
 MathType@MTEF@5@5@+=feaafiart1ev1aaatCvAUfKttLearuWrP9MDH5MBPbIqV92AaeXatLxBI9gBaebbnrfifHhDYfgasaacH8akY=wiFfYdH8Gipec8Eeeu0xXdbba9frFj0=OqFfea0dXdd9vqai=hGuQ8kuc9pgc9s8qqaq=dirpe0xb9q8qiLsFr0=vr0=vr0dc8meaabaqaciaacaGaaeqabaqabeGadaaakeaacqWGubavdaWgaaWcbaacciGae8xSde2aaSbaaWqaaiabikdaYaqabaaaleqaaaaa@30D9@ in addition to the Bayesian score difference (5), and tends to produce more balanced trees. In the final step, we need to cut the hierarchical tree *T*_0_. To this end we traverse the tree from the root towards its leaves. If we are at a subtree node *T_α _*with children Tα1
 MathType@MTEF@5@5@+=feaafiart1ev1aaatCvAUfKttLearuWrP9MDH5MBPbIqV92AaeXatLxBI9gBaebbnrfifHhDYfgasaacH8akY=wiFfYdH8Gipec8Eeeu0xXdbba9frFj0=OqFfea0dXdd9vqai=hGuQ8kuc9pgc9s8qqaq=dirpe0xb9q8qiLsFr0=vr0=vr0dc8meaabaqaciaacaGaaeqabaqabeGadaaakeaacqWGubavdaWgaaWcbaacciGae8xSde2aaSbaaWqaaiabigdaXaqabaaaleqaaaaa@30D7@ and Tα2
 MathType@MTEF@5@5@+=feaafiart1ev1aaatCvAUfKttLearuWrP9MDH5MBPbIqV92AaeXatLxBI9gBaebbnrfifHhDYfgasaacH8akY=wiFfYdH8Gipec8Eeeu0xXdbba9frFj0=OqFfea0dXdd9vqai=hGuQ8kuc9pgc9s8qqaq=dirpe0xb9q8qiLsFr0=vr0=vr0dc8meaabaqaciaacaGaaeqabaqabeGadaaakeaacqWGubavdaWgaaWcbaacciGae8xSde2aaSbaaWqaaiabikdaYaqabaaaleqaaaaa@30D9@, we compute the score difference (5). If this difference is negative, the total score is improved by keeping the split *T_α_*, and we move on to test each of its children nodes. If the difference is positive, the total score is improved by not making the split *T_α_*, and we remove its children nodes from the tree. The experiment set ℰ
 MathType@MTEF@5@5@+=feaafiart1ev1aaatCvAUfKttLearuWrP9MDH5MBPbIqV92AaeXatLxBI9gBamrtHrhAL1wy0L2yHvtyaeHbnfgDOvwBHrxAJfwnaebbnrfifHhDYfgasaacH8akY=wiFfYdH8Gipec8Eeeu0xXdbba9frFj0=OqFfea0dXdd9vqai=hGuQ8kuc9pgc9s8qqaq=dirpe0xb9q8qiLsFr0=vr0=vr0dc8meaabaqaciaacaGaaeqabaWaaeGaeaaakeaaimaacqWFWesraaa@3785@_*α *_becomes one of the leaves of the regulation program, contributing one term in the sum (4).

The pseudocode for the regulation program learning algorithm is given in Figure S3 in Additional file 1. In [6,30], regulation programs are learned top-down by considering all possible splits on all current leaves with all potential regulators, so regulation trees and regulator assignments are learned simultaneously. As a result missing regulators or noise in the regulator data might lead to a suboptimal partitioning of the experiments in a module. In our approach we have focused on finding an optimal partition of the module regardless of the set of potential regulators. A module collects the data of many genes and therefore this partition will be less affected by noise or missing data than when it is determined by exact splits on single regulators.

### Regulator assignment

At a given internal node *T_α _*of the regulation tree *T*_0_, the experiment set ℰ
 MathType@MTEF@5@5@+=feaafiart1ev1aaatCvAUfKttLearuWrP9MDH5MBPbIqV92AaeXatLxBI9gBamrtHrhAL1wy0L2yHvtyaeHbnfgDOvwBHrxAJfwnaebbnrfifHhDYfgasaacH8akY=wiFfYdH8Gipec8Eeeu0xXdbba9frFj0=OqFfea0dXdd9vqai=hGuQ8kuc9pgc9s8qqaq=dirpe0xb9q8qiLsFr0=vr0=vr0dc8meaabaqaciaacaGaaeqabaWaaeGaeaaakeaaimaacqWFWesraaa@3785@_*α *_is partitioned into two distinct sets ℰα1
 MathType@MTEF@5@5@+=feaafiart1ev1aaatCvAUfKttLearuWrP9MDH5MBPbIqV92AaeXatLxBI9gBaebbnrfifHhDYfgasaacH8akY=wiFfYdH8Gipec8Eeeu0xXdbba9frFj0=OqFfea0dXdd9vqai=hGuQ8kuc9pgc9s8qqaq=dirpe0xb9q8qiLsFr0=vr0=vr0dc8meaabaqaciaacaGaaeqabaqabeGadaaakeaat0uy0HwzTfgDPnwy1egaryqtHrhAL1wy0L2yHvdaiqaacqWFWesrdaWgaaWcbaacciGae4xSde2aaSbaaWqaaiabigdaXaqabaaaleqaaaaa@3A7D@ and ℰα2
 MathType@MTEF@5@5@+=feaafiart1ev1aaatCvAUfKttLearuWrP9MDH5MBPbIqV92AaeXatLxBI9gBaebbnrfifHhDYfgasaacH8akY=wiFfYdH8Gipec8Eeeu0xXdbba9frFj0=OqFfea0dXdd9vqai=hGuQ8kuc9pgc9s8qqaq=dirpe0xb9q8qiLsFr0=vr0=vr0dc8meaabaqaciaacaGaaeqabaqabeGadaaakeaat0uy0HwzTfgDPnwy1egaryqtHrhAL1wy0L2yHvdaiqaacqWFWesrdaWgaaWcbaacciGae4xSde2aaSbaaWqaaiabikdaYaqabaaaleqaaaaa@3A7F@ according to the tree structure. Given a regulator *r *and split value *s*, we can also partition ℰ
 MathType@MTEF@5@5@+=feaafiart1ev1aaatCvAUfKttLearuWrP9MDH5MBPbIqV92AaeXatLxBI9gBamrtHrhAL1wy0L2yHvtyaeHbnfgDOvwBHrxAJfwnaebbnrfifHhDYfgasaacH8akY=wiFfYdH8Gipec8Eeeu0xXdbba9frFj0=OqFfea0dXdd9vqai=hGuQ8kuc9pgc9s8qqaq=dirpe0xb9q8qiLsFr0=vr0=vr0dc8meaabaqaciaacaGaaeqabaWaaeGaeaaakeaaimaacqWFWesraaa@3785@_*α *_into two sets

ℛ1={m∈ℰα:xr,m≤s}ℛ2={m∈ℰα:xr,m>s},
 MathType@MTEF@5@5@+=feaafiart1ev1aaatCvAUfKttLearuWrP9MDH5MBPbIqV92AaeXatLxBI9gBamrtHrhAL1wy0L2yHvtyaeHbnfgDOvwBHrxAJfwnaebbnrfifHhDYfgasaacH8akY=wiFfYdH8Gipec8Eeeu0xXdbba9frFj0=OqFfea0dXdd9vqai=hGuQ8kuc9pgc9s8qqaq=dirpe0xb9q8qiLsFr0=vr0=vr0dc8meaabaqaciaacaGaaeqabaWaaeGaeaaakeaafaqabeGabaaabaacdaGae83gHi1aaSbaaSqaaiabigdaXaqabaGccqGH9aqpcqGG7bWEcqWGTbqBcqGHiiIZcqWFWesrdaWgaaWcbaacciGae4xSdegabeaakiabcQda6iabdIha4naaBaaaleaacqWGYbGCcqGGSaalcqWGTbqBaeqaaOGaeyizImQaem4CamNaeiyFa0habaGae83gHi1aaSbaaSqaaiabikdaYaqabaGccqGH9aqpcqGG7bWEcqWGTbqBcqGHiiIZcqWFWesrdaWgaaWcbaGae4xSdegabeaakiabcQda6iabdIha4naaBaaaleaacqWGYbGCcqGGSaalcqWGTbqBaeqaaOGaeyOpa4Jaem4CamNaeiyFa0NaeiilaWcaaaaa@61D6@

where *x*_*r*,*m *_is the expression value of regulator *r *in experiment *m*.

Consider now two random variables: *E *which can take the values *α*_1 _or *α*_2_, and *R *which can take the values 1 or 2, with probabilities defined by simple counting, *p*(*E *= *α*_1_) = |ℰα1
 MathType@MTEF@5@5@+=feaafiart1ev1aaatCvAUfKttLearuWrP9MDH5MBPbIqV92AaeXatLxBI9gBaebbnrfifHhDYfgasaacH8akY=wiFfYdH8Gipec8Eeeu0xXdbba9frFj0=OqFfea0dXdd9vqai=hGuQ8kuc9pgc9s8qqaq=dirpe0xb9q8qiLsFr0=vr0=vr0dc8meaabaqaciaacaGaaeqabaqabeGadaaakeaat0uy0HwzTfgDPnwy1egaryqtHrhAL1wy0L2yHvdaiqaacqWFWesrdaWgaaWcbaacciGae4xSde2aaSbaaWqaaiabigdaXaqabaaaleqaaaaa@3A7D@|/|ℰ
 MathType@MTEF@5@5@+=feaafiart1ev1aaatCvAUfKttLearuWrP9MDH5MBPbIqV92AaeXatLxBI9gBamrtHrhAL1wy0L2yHvtyaeHbnfgDOvwBHrxAJfwnaebbnrfifHhDYfgasaacH8akY=wiFfYdH8Gipec8Eeeu0xXdbba9frFj0=OqFfea0dXdd9vqai=hGuQ8kuc9pgc9s8qqaq=dirpe0xb9q8qiLsFr0=vr0=vr0dc8meaabaqaciaacaGaaeqabaWaaeGaeaaakeaaimaacqWFWesraaa@3785@_*α*_|, *p*(*R *= 1) = |ℛ
 MathType@MTEF@5@5@+=feaafiart1ev1aaatCvAUfKttLearuWrP9MDH5MBPbIqV92AaeXatLxBI9gBamrtHrhAL1wy0L2yHvtyaeHbnfgDOvwBHrxAJfwnaebbnrfifHhDYfgasaacH8akY=wiFfYdH8Gipec8Eeeu0xXdbba9frFj0=OqFfea0dXdd9vqai=hGuQ8kuc9pgc9s8qqaq=dirpe0xb9q8qiLsFr0=vr0=vr0dc8meaabaqaciaacaGaaeqabaWaaeGaeaaakeaaimaacqWFBeIuaaa@377D@_1_|/|ℰ
 MathType@MTEF@5@5@+=feaafiart1ev1aaatCvAUfKttLearuWrP9MDH5MBPbIqV92AaeXatLxBI9gBamrtHrhAL1wy0L2yHvtyaeHbnfgDOvwBHrxAJfwnaebbnrfifHhDYfgasaacH8akY=wiFfYdH8Gipec8Eeeu0xXdbba9frFj0=OqFfea0dXdd9vqai=hGuQ8kuc9pgc9s8qqaq=dirpe0xb9q8qiLsFr0=vr0=vr0dc8meaabaqaciaacaGaaeqabaWaaeGaeaaakeaaimaacqWFWesraaa@3785@_*α*_|, etc. We are interested in the uncertainty in *E *given knowledge (through the data) of *R*, i.e., in the conditional entropy [32]

*H*(*E *| *R*) = *p*_1_*h*(*q*_1_) + *p*_2_*h*(*q*_2_),     (6)

where *p*_*i *_= *p*(*R *= *i*), *h *is the binary entropy function

*h*(*q*) = -*q *log(*q*) - (1 - *q*) log(1 - *q*),

and *q*_*i *_are the conditional probabilities

qi=p(E=α1|R=i)=|ℰα1∩ℛi||ℛi|,i=1,2.
 MathType@MTEF@5@5@+=feaafiart1ev1aaatCvAUfKttLearuWrP9MDH5MBPbIqV92AaeXatLxBI9gBaebbnrfifHhDYfgasaacH8akY=wiFfYdH8Gipec8Eeeu0xXdbba9frFj0=OqFfea0dXdd9vqai=hGuQ8kuc9pgc9s8qqaq=dirpe0xb9q8qiLsFr0=vr0=vr0dc8meaabaqaciaacaGaaeqabaqabeGadaaakeaafaqabeqacaaabaGaemyCae3aaSbaaSqaaiabdMgaPbqabaGccqGH9aqpcqWGWbaCcqGGOaakcqWGfbqrcqGH9aqpiiGacqWFXoqydaWgaaWcbaGaeGymaedabeaakiabcYha8jabdkfasjabg2da9iabdMgaPjabcMcaPiabg2da9maalaaabaGaeiiFaW3enfgDOvwBHrxAJfwnHbqeg0uy0HwzTfgDPnwy1aaceaGae4hmHu0aaSbaaSqaaiab=f7aHnaaBaaameaacqaIXaqmaeqaaaWcbeaakiabgMIihlab+TrisnaaBaaaleaacqWGPbqAaeqaaOGaeiiFaWhabaGaeiiFaWNae43gHi1aaSbaaSqaaiabdMgaPbqabaGccqGG8baFaaGaeiilaWcabaGaemyAaKMaeyypa0JaeGymaeJaeiilaWIaeGOmaiJaeiOla4caaaaa@6079@

In the presence of missing data, the probabilities *p*_*i *_and *q*_*i *_need to be modified to take into account this extra uncertainty, details are given in Additional file 1.

The conditional entropy is nonnegative and reaches its minimum value 0 when *q*_1 _= 0 or 1 (and consequently *q*_2 _= 1, resp. 0), which means the ℰ
 MathType@MTEF@5@5@+=feaafiart1ev1aaatCvAUfKttLearuWrP9MDH5MBPbIqV92AaeXatLxBI9gBamrtHrhAL1wy0L2yHvtyaeHbnfgDOvwBHrxAJfwnaebbnrfifHhDYfgasaacH8akY=wiFfYdH8Gipec8Eeeu0xXdbba9frFj0=OqFfea0dXdd9vqai=hGuQ8kuc9pgc9s8qqaq=dirpe0xb9q8qiLsFr0=vr0=vr0dc8meaabaqaciaacaGaaeqabaWaaeGaeaaakeaaimaacqWFWesraaa@3785@ and ℛ
 MathType@MTEF@5@5@+=feaafiart1ev1aaatCvAUfKttLearuWrP9MDH5MBPbIqV92AaeXatLxBI9gBamrtHrhAL1wy0L2yHvtyaeHbnfgDOvwBHrxAJfwnaebbnrfifHhDYfgasaacH8akY=wiFfYdH8Gipec8Eeeu0xXdbba9frFj0=OqFfea0dXdd9vqai=hGuQ8kuc9pgc9s8qqaq=dirpe0xb9q8qiLsFr0=vr0=vr0dc8meaabaqaciaacaGaaeqabaWaaeGaeaaakeaaimaacqWFBeIuaaa@377D@ partitions are equal and the regulator – split value pair 'explains' the split in the regulation tree exactly. Hence we assign to each internal node of a regulation tree the regulator – split value pair which minimizes the conditional entropy (6). Since this assignment has to be done only once, after the module networks score has converged, the best regulator – split value pairs can be found by simply enumerating over all possibilities, even for relatively large data sets. The actual algorithm for assigning regulators to all nodes operates first on nodes closer to the roots of the trees where the most significant splits are located, and takes into account acyclicity constraints on the module network. It is presented in pseudocode in Figure S4 in Additional file 1.

### Learning module networks

To find an optimal module network, learning of regulation trees is alternated with reassigning genes to other modules until convergence of the Bayesian score. Module initialization can be done using any clustering algorithm. Here, we used *k*-means [33], and reassigning is done like in [30] by making all single-gene moves from one module to another which improve the total score.

### Network comparison

To obtain a gene network from a module network, we put directed edges from the regulators of a module to all the genes in that module. We compare inferred to true network by computing the number of edges that are true positive (tp), false positive (fp) and false negative (fn). Standard measures for the inference quality are precision and recall. Precision (denoted *P*) is defined as the fraction of edges in the inferred module network that is correct, and recall (denoted *R*) as the fraction of edges in the true network that is correctly inferred, i.e.,

P=tptp+fpR=tptp+fn.
 MathType@MTEF@5@5@+=feaafiart1ev1aaatCvAUfKttLearuWrP9MDH5MBPbIqV92AaeXatLxBI9gBaebbnrfifHhDYfgasaacH8akY=wiFfYdH8Gipec8Eeeu0xXdbba9frFj0=OqFfea0dXdd9vqai=hGuQ8kuc9pgc9s8qqaq=dirpe0xb9q8qiLsFr0=vr0=vr0dc8meaabaqaciaacaGaaeqabaqabeGadaaakeaafaqabeqacaaabaGaemiuaaLaeyypa0ZaaSaaaeaaieaacqWF0baDcqWFWbaCaeaacqWF0baDcqWFWbaCcqGHRaWkcqWFMbGzcqWFWbaCaaaabaGaemOuaiLaeyypa0ZaaSaaaeaacqWF0baDcqWFWbaCaeaacqWF0baDcqWFWbaCcqGHRaWkcqWFMbGzcqWFUbGBaaaaaiabc6caUaaa@449C@

The *F*-measure, defined as the harmonic mean of precision and recall, F=2PRP+R
 MathType@MTEF@5@5@+=feaafiart1ev1aaatCvAUfKttLearuWrP9MDH5MBPbIqV92AaeXatLxBI9gBaebbnrfifHhDYfgasaacH8akY=wiFfYdH8Gipec8Eeeu0xXdbba9frFj0=OqFfea0dXdd9vqai=hGuQ8kuc9pgc9s8qqaq=dirpe0xb9q8qiLsFr0=vr0=vr0dc8meaabaqaciaacaGaaeqabaqabeGadaaakeaacqWGgbGrcqGH9aqpdaWcaaqaaiabikdaYiabdcfaqjabdkfasbqaaiabdcfaqjabgUcaRiabdkfasbaaaaa@3557@, can be used as a single measure for inference quality.

The module content for different module networks can be compared by computing for each module in one network how many genes of it are also grouped together in one module in the other network, and averaging over the number of modules. We call this the average module overlap.

### GO overrepresentation analysis

GO enrichment *P*-values for all modules were determined using the BiNGO tool [34], which was incorporated into the LeMoNe package. The overrepresentation of GO Biological Process categories was tested using hypergeometric tests and the resulting *P*-values were corrected for multiple testing using a False Discovery Rate correction.

### Software

The latest version of SynTReN can be downloaded from [35] and the latest version of Genomica from [27]. LeMoNe is implemented in Java and available for download in source or executable form [36].

## Results and discussion

### Implementation differences in LeMoNe versus Genomica

As a starting point for the development of LeMoNe, we re-implemented the methodology described by Segal *et al*. [6], which is incorporated in the Genomica software package. Briefly, Genomica takes as input a gene expression data set and a list of potential regulators. After an initial clustering step, the algorithm iteratively constructs a regulatory program for each of the modules (clusters) in the form of a regression tree, and then reassigns each gene to the module whose program best predicts the gene's expression behavior. These two steps are repeated until convergence is reached. In this process, the algorithm attempts to maximize a Bayesian score function that evaluates the model's fit to the data [6].

We used the same overall strategy and the same Bayesian score function in LeMoNe. However, with respect to the original methods described by Segal *et al*. [6], LeMoNe incorporates an alternative strategy for inferring regulatory programs that offers some advantages (see Methods). First, LeMoNe uses a Bayesian hierarchical clustering strategy to learn the regulation trees for the modules from the bottom up instead of from the top down. Furthermore, contrary to Genomica [6], the partitioning of expression data inside a module is not dependent on the expression profiles of the potential regulators, but only on the module data itself. This should allow the program to better handle missing or 'hidden' regulators (see further). As an additional advantage, the assignment of regulators to regulation program nodes can be postponed until after the final convergence of the Bayesian score, which leads to considerable time savings (see further).

A second modification in LeMoNe is that regulators are assigned to the splits in the regulation tree (data splits) based on an information theoretic measure, namely the conditional entropy of the partition of the regulator's expression profile dictated by the data split, given the partition imposed by a particular split value (see Methods). As a consequence, a data split does not impose, but merely prefers, a clean partition of the best-matching regulator's expression values around a certain split value. In comparison with Genomica, where only such clean partitions are used, this strategy has the advantage that potential noise in the regulator's expression is taken into account. Additionally, the conditional entropy can be used to estimate the quality of the regulator assignment, and thus suggest missing potential regulators for splits without a low-entropy regulator. Information theory has been used before to analyze and cluster gene expression data [13,22-26]. Our method introduces elements of information theory into the module networks formalism.

In the following sections, we use SynTReN data to test LeMoNe in a completely controlled situation in which simulated microarray data is analyzed for a known underlying regulatory network of reasonable size, and we assess the performance effects of the aforementioned methodological changes with respect to Genomica [6]. The LeMoNe package and the source code are freely available under the GPL license (see Software section).

### Modularity

A fundamental assumption of the module networks formalism is that real biological networks have a modular structure [18] that is reflected in the gene expression data, and therefore groups of genes can share the same parameters in the mathematical description of the network. In LeMoNe, as in other module network learning programs [6,11], the desired number of modules has to be given as an input parameter to the inference program, and a main question is how the optimal module number has to be determined.

Fewer modules means lower computational cost and more data points per module. This results in a better estimation of parameters, but possibly entails oversimplifying the network and missing important regulatory relations. More modules means more specific optimization of the network at higher computational cost. When modules become too small, there could be too few data points per module for a reliable estimation of the parameters. In this section we use the Bayesian score to estimate the optimal number of modules.

**Figure 2:**
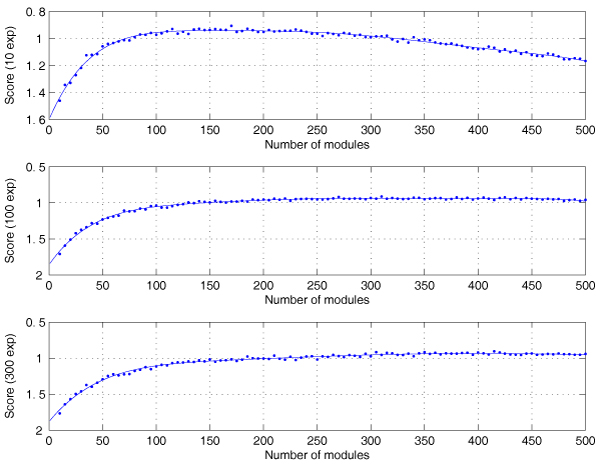
**Bayesian score as a function of the number of modules and experiments**. Bayesian score as a function of the number of modules for data sets with 10, 100 and 300 experiments (top to bottom). The score is normalized by the number of genes times the number of experiments. The curves are least squares fits of the data to a linear non-polynomial model of the form a0+∑k=1nakxk−1e−x/500
 MathType@MTEF@5@5@+=feaafiart1ev1aaatCvAUfKttLearuWrP9MDH5MBPbIqV92AaeXatLxBI9gBaebbnrfifHhDYfgasaacH8akY=wiFfYdH8Gipec8Eeeu0xXdbba9frFj0=OqFfea0dXdd9vqai=hGuQ8kuc9pgc9s8qqaq=dirpe0xb9q8qiLsFr0=vr0=vr0dc8meaabaqaciaacaGaaeqabaqabeGadaaakeaacqWGHbqydaWgaaWcbaGaeGimaadabeaakiabgUcaRmaaqadabaGaemyyae2aaSbaaSqaaiabdUgaRbqabaGccqWG4baEdaahaaWcbeqaaiabdUgaRjabgkHiTiabigdaXaaakiabdwgaLnaaCaaaleqabaGaeyOeI0IaemiEaGNaei4la8IaeGynauJaeGimaaJaeGimaadaaaqaaiabdUgaRjabg2da9iabigdaXaqaaiabd6gaUbqdcqGHris5aaaa@461A@ with *x *the number of modules and *n *= 6.

Throughout this manuscript, we make use of a SynTReN-generated synthetic network encompassing 1000 genes of which 105 act as regulators (see Methods). Unless otherwise stated, we use all 105 regulators in this network as potential regulators while inferring module networks. Figure 2 shows the Bayesian score, normalized by the number of genes times the number of experiments, for this network and different numbers of experiments. In all three panels, the score reaches a maximum. The top panel (data set with 10 experiments), which has a true maximum for the score, illustrates that the network inference problem is underdetermined for very small data sets. Increasing the number of modules beyond the location of the maximum lowers the fit of the model to the data. For larger data sets (middle and bottom panel, 100, resp. 300 experiments), the score saturates and after a certain point the model does not improve anymore by increasing the number of modules. Hence, the optimal number of modules should be situated around the point where the Bayesian score starts to level off. For increasing number of experiments, the optimal number shifts to the right. This suggests that increasing amounts of data enable the algorithm to uncover smaller and more finetuned modules. However, the rightbound shift of the optimum becomes less pronounced for increasing number of experiments. This reflects the fact that only a limited number of modules are inherently present in the true network.

We define the number of modules in the true network as the number of gene sets having the same set of regulators (taking into account activator or repressor type). This number is 286 for the 1000 gene synthetic network we consider here, among which there are 180 with at least 3 genes and 126 with at least 5 genes. The saturation behavior of the score curves for 100 and 300 experiments in Figure 2 more or less reflects the modularity in the true network.

### Network inference performance

A more detailed analysis of network inference performance is obtained by comparing the set of regulator to gene edges in the true (synthetic) network and in the inferred module network. We use standard measures such as recall, precision, and *F*-measure (see Methods).

**Figure 3:**
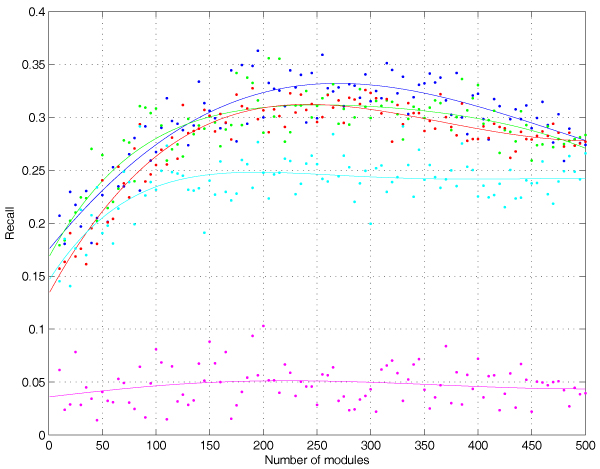
**Recall as a function of the number of modules and experiments**. Recall as a function of the number of modules for data sets with 10 (magenta), 50 (cyan), 100 (red), 200 (green), and 300 (blue) experiments. The curves are least squares fits of the data to a linear non-polynomial model of the form a0+∑k=1nakxk−1e−x/500
 MathType@MTEF@5@5@+=feaafiart1ev1aaatCvAUfKttLearuWrP9MDH5MBPbIqV92AaeXatLxBI9gBaebbnrfifHhDYfgasaacH8akY=wiFfYdH8Gipec8Eeeu0xXdbba9frFj0=OqFfea0dXdd9vqai=hGuQ8kuc9pgc9s8qqaq=dirpe0xb9q8qiLsFr0=vr0=vr0dc8meaabaqaciaacaGaaeqabaqabeGadaaakeaacqWGHbqydaWgaaWcbaGaeGimaadabeaakiabgUcaRmaaqadabaGaemyyae2aaSbaaSqaaiabdUgaRbqabaGccqWG4baEdaahaaWcbeqaaiabdUgaRjabgkHiTiabigdaXaaakiabdwgaLnaaCaaaleqabaGaeyOeI0IaemiEaGNaei4la8IaeGynauJaeGimaaJaeGimaadaaaqaaiabdUgaRjabg2da9iabigdaXaqaaiabd6gaUbqdcqGHris5aaaa@461A@ with *x *the number of modules and *n *= 3.

Figure 3 shows the recall as a function of the number of modules for different numbers of experiments. The location of the recall maxima seems to agree well with the saturation points of the corresponding Bayesian score curves (Figure 2). As expected the maximal recall, and hence the total number of true positives, increases for data sets with more experiments, saturating between 30 and 35% for data sets with ≥100 experiments.

A similar saturation with increasing number of experiments is seen for the precision curves (Figure S1 in Additional file 1) and the *F*-measure curves (Figure S2 in Additional file 1). Whereas the precision continues to increase with the number of modules, the *F*-measure saturates, but does so at a higher number of modules than the Bayesian score. Taking into account the modular composition of the true network (see previous section), the Bayesian score and the recall curves seem to generate better estimates of the optimal number of modules than the *F*-measure curves.

**Figure 4:**
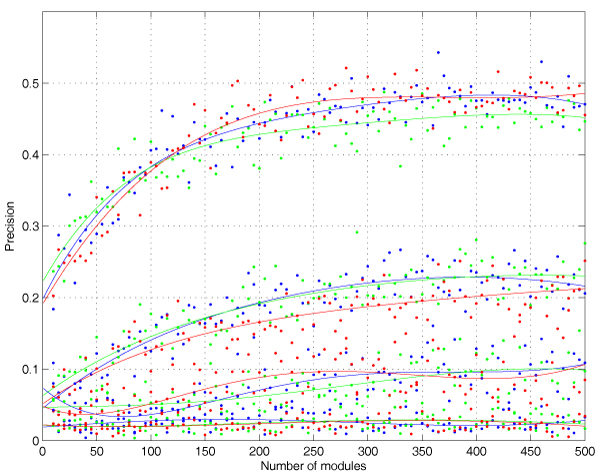
**Precision at different regulation tree levels**. Precision as a function of the number of modules for subnetworks generated by regulation tree levels 0 (roots), 1 and 2, and for random assignments of regulators to regulation tree nodes (top to bottom) for data sets with 100 (red), 200 (green) and 300 (blue) experiments. The curves are least squares fits of the data to a linear non-polynomial model of the form a0+∑k=1nakxk−1e−x/500
 MathType@MTEF@5@5@+=feaafiart1ev1aaatCvAUfKttLearuWrP9MDH5MBPbIqV92AaeXatLxBI9gBaebbnrfifHhDYfgasaacH8akY=wiFfYdH8Gipec8Eeeu0xXdbba9frFj0=OqFfea0dXdd9vqai=hGuQ8kuc9pgc9s8qqaq=dirpe0xb9q8qiLsFr0=vr0=vr0dc8meaabaqaciaacaGaaeqabaqabeGadaaakeaacqWGHbqydaWgaaWcbaGaeGimaadabeaakiabgUcaRmaaqadabaGaemyyae2aaSbaaSqaaiabdUgaRbqabaGccqWG4baEdaahaaWcbeqaaiabdUgaRjabgkHiTiabigdaXaaakiabdwgaLnaaCaaaleqabaGaeyOeI0IaemiEaGNaei4la8IaeGynauJaeGimaaJaeGimaadaaaqaaiabdUgaRjabg2da9iabigdaXaqaaiabd6gaUbqdcqGHris5aaaa@461A@ with *x *the number of modules and *n *= 3.

We also investigated whether the inferred regulation programs provide any information regarding the quality of the regulators. When analyzing real data, such information could be useful to prioritize regulators for experimental validation. A first property which we tried to relate to a regulator's quality is its hierarchical location in the regulation program. It seems that regulators deeper in the regulation tree become progressively less relevant. Figure 4 illustrates this effect by showing separately the precisions for the roots of the regulation trees (level 0), the children of the roots (level 1), and the grandchildren (level 2) for data sets with 100, 200, and 300 experiments. The precisions for the various regulatory levels remain within each others standard deviation across the tested range of experiments, but the precision clearly diminishes with increasing levels in the regulation program. For each data set and inferred module network we created an additional network where each module is assigned a random regulator set of the same size as in the inferred network. The precision for these random regulation programs is shown in the bottom most curves in Figure 4. For regulation levels beyond level 2, the precisions fall in this region of random assignments and they add almost exclusively false positives (results not shown). In general, we can say that the top regulators are far more likely to represent true regulatory interactions.

**Figure 5:**
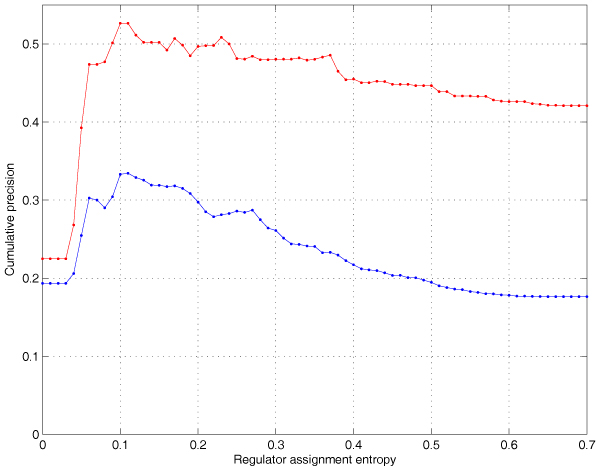
**Cumulative distribution of precision as a function of regulator entropy**. Cumulative distribution of precision as a function of regulator entropy for the data set with 100 experiments and 150 modules: each point at an entropy value *x *(spaced at 0.01 intervals) gives the precision of all (blue) or top (red) regulators with assignment entropy ≤*x*.

An additional layer of information is provided by the regulator assignment entropies. A low value of the entropy corresponds to a regulator matching well with a split in the expression pattern of the regulated module. Hence we expect regulators with low entropy to have a higher probability to be true regulators. This is illustrated in Figure 5. For the data set with 100 experiments and 150 modules, the subnetwork generated by all regulators with an entropy lower than, e.g., 0.1 has precision 0.334, almost twice as high as the precision of 0.176 for the whole module network. For the subnetwork generated by the regulators at the roots of the regulation trees, the precision increases from 0.42 to 0.53 by introducing the same entropy cut-off. Other data sets show similar behavior (data not shown).

### Performance of LeMoNe versus Genomica

**Figure 6:**
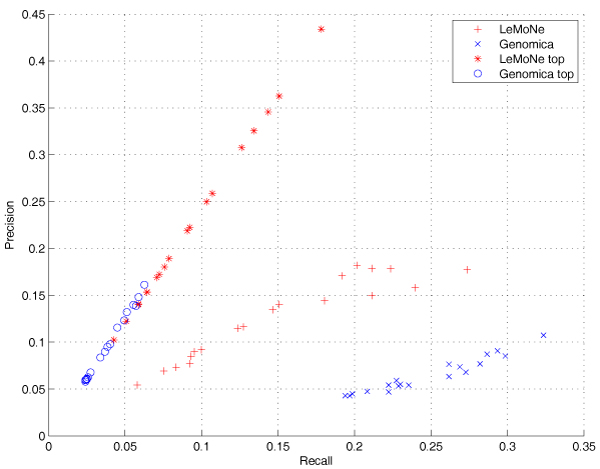
**Comparison of heuristic search methods**. Comparison of heuristic search methods by recall – precision pairs for data sets with 100 experiments and different noise levels, for the complete module network, and for the subnetwork generated by the top regulators in the regulation programs.

Next, we compared the performance of LeMoNe and Genomica [6,27]. Both programs heuristically search for an optimal module network and are therefore bound to end up at a (different) local maximum of the Bayesian score. We simulated 10 different data sets with 100 experiments for the same 1000 gene network as before and inferred a network with 150 modules (corresponding to the point where the score function in Figure 2 starts to saturate). The average precisions are 0.196 ± 0.015, resp. 0.155 ± 0.013, and average recalls 0.255 ± 0.016, resp. 0.381 ± 0.021, for LeMoNe, resp. Genomica. The average *F*-measure is 0.222 ± 0.015, resp. 0.220 ± 0.016. The similarity in performance at the level of the whole module network, with a bias for higher precision in LeMoNe and higher recall in Genomica, is further seen in Figure 6, where we plot recall – precision pairs for both programs at different noise levels. For each of the plotted series, lower noise levels correspond to points in the upper right of the series plot, and higher noise levels to points in the bottom left, illustrating a general decrease in performance for more noisy data.

The average module overlap between the module networks generated by LeMoNe and Genomica is 0.46 ± 0.02. Both programs, although featuring similar performance, attain a different local maximum of the Bayesian score, and the differences in the corresponding module networks can be quite substantial. In general we can say that both module network inference programs suffer from a high number of false positive edges. When using LeMoNe, false positives can to some extent be filtered out by looking only at the highest levels in the regulation tree (Figure 4). To see whether this is also the case for Genomica, we calculated the recall and precision for the subnetworks generated by the top regulators alone (Figure 6).

The recall for these subnetworks is generally lower as they contain far fewer edges than the complete module network. For LeMoNe this decrease in recall is compensated by a large increase in precision. For Genomica the decrease in recall is bigger, with only a slight increase in precision. There is no analogue of the assignment entropy in Genomica, so we cannot compare the gain in precision by imposing an entropy cut-off.

One of the major differences in LeMoNe with respect to Genomica is the fact that the regulatory tree structures learned by LeMoNe are only dependent on the expression data inside the module, and not on the expression profiles of potential regulators. We hypothesized that this might allow LeMoNe to better handle missing or hidden regulators, a situation which might for instance occur if the true regulator is missing from the list of potential regulators. In order to test this hypothesis, we simulated 10 different data sets with 100 experiments for the same 1000 gene network and inferred module networks with 150 modules using both LeMoNe and Genomica. In each of the ten runs we randomly left out 20% of the potential regulators from the regulator list (i.e., we used 84 instead of 105 potential regulators). The average *F*-measure of the resulting networks is 0.183 ± 0.025 for LeMoNe, versus 0.126 ± 0.012 for Genomica. Compared to the results when taking into account all 105 potential regulators (see above), the performance drop for LeMoNe is clearly less pronounced (17.6%) than for Genomica (42.7%), indicating that LeMoNe is indeed better at handling missing regulators.

Regarding the speed of LeMoNe versus Genomica, we can say that LeMoNe is considerably faster for larger data sets. This is mainly due to the fact that in LeMoNe the regulators need only be assigned to the regulation programs once, after the final convergence of the Bayesian score. This saves a considerable amount of time on scanning possible split values and performing acyclicity checks at each iteration. Roughly, LeMoNe and Genomica performed equally in terms of speed on the SynTReN data set containing 1000 genes and 100 experiments. On a real data set with 173 experiments [21], LeMoNe was about twice as fast as Genomica when limiting the number of genes to 1000, and ten times faster when considering the whole data set (2355 genes).

### Biological data

For real biological data sets the underlying regulatory network is generally not known (indeed, the primary purpose of module network learning algorithms is precisely to infer the regulatory network) and hence it is difficult to assess the quality of an inferred network. This is one of the main reasons why microarray data simulators such as SynTReN have to be used to validate the methodology. However, given the fact that data simulators seldom capture all aspects of real biological systems, any results obtained on simulated data should be approached critically and, where possible, validated on biological data sets. Here, we investigate to what extent module networks inferred from real expression data can be validated using structured biological information.

For *S. cerevisiae*, there is partial information on the underlying network structure in the form of ChIP-chip data and promoter motif data [9], and more profusely in the form of GO annotations [2]. We learned module networks for budding yeast from an expression compendium containing data for 2355 genes under 173 different stress conditions [21] (the Gasch data set) using the same number of modules (50) and the same list of potential regulators as Segal *et al*. [6]. We then calculated the *F*-measure between the resulting regulatory network and the ChIP-chip network of Harbison *et al*. [9], considering in the former network only regulators that were tested by ChIP-chip. In general, the resulting recall and precision values are substantially lower than for simulated data of the same size, namely 0.0195, resp 0.0218. When looking at individual modules, only 13 out of 50 regulatory programs feature at least one regulator that is to some extent confirmed by ChIP-chip data. In addition, we tried to relate the regulatory program of a module to the module's gene content in functional terms using GO annotation. Overall, only 8 out of 50 programs possess one or more regulators belonging to a yeast GOSlim Biological Process category that is overrepresented in the module (considering only the leaf categories in the GOSlim hierarchy). Remarkably, only 3 of these 8 programs overlap with the 13 regulatory programs featuring overlap with the ChIP-chip data. This observation suggests that both data types can actually be used only to a limited extent to infer the quality of regulation programs. Indeed, many factors limit the use of ChIP-chip and GO data as 'gold standards'. Both types of data are noisy and offer incomplete information. For example, Harbison *et al*. [9] mainly profiled transcription factor binding in rich medium conditions, whereas the Gasch data set contains primarily stress conditions. The parts of the transcriptional network that are active under these conditions may substantially differ [9,10]. Moreover, the expression profile of a transcription factor is often not directly related to the expression profile of its targets, for example due to post-translational regulation of transcription factor activity. As a consequence, indirect regulators such as upstream signal transducers may feature in the regulation programs instead of the direct regulators, i.e., the transcription factors [6].

As for GO, many regulators appear not to be annotated to the GO Biological Process categories of their target genes. Taking these factors into account, the limited overlap with the available ChIP-chip and GO data does not necessarily reflect the quality of the inferred regulatory programs.

On the contrary, we established that the regulatory programs do in fact contain a considerable amount of relevant and potentially valuable information. Indeed, by manually investigating individual modules in more detail, we could in many cases qualitatively relate the regulators to the module's gene content. For example, the module shown in Figure 1 is enriched in a.o. genes involved in the main pathways of carbohydrate metabolism (*P *= 1.0596E - 4), energy derivation by oxidation of organic compounds (*P *= 1.2046E - 4) and alcohol biosynthesis (*P *= 1.3185E - 2). None of the 5 regulators of this module could be related to the module's gene content based on ChIP-chip or GO information. However, based on their description in the *Saccharomyces *Genome Database (SGD) [28], all 5 regulators could be linked to glucose sensing or the response to (glucose) starvation, processes that can arguably influence the expression of carbohydrate metabolism genes.

However, one must keep in mind that it remains impossible to infer complete and accurate regulatory networks from gene expression data alone. Expression data only provides information on one regulatory level, namely the transcriptional level. Information on (post-)translational regulation is lacking. The current expression-based module network algorithms (e.g. [6], this study) try to remedy this problem by including signal transducers in the list of potential regulators in addition to transcription factors, in the hope to capture some of this non-transcriptional regulation from the expression profiles of key signal transducers. However, this trick can only be expected to uncover a fraction of such non-transcriptional regulatory interactions, and moreover the direct targets of these regulatory interactions are not identified. A potential remedy for this shortcoming would be to include other types of data, such as data on protein expression levels and protein phosphorylation, in the module network learning framework. Unfortunately, such data are not yet available on a large scale.

In summary, our results indicate that structured biological information such as ChIP-chip data or GO can not (yet) be used to measure the performance of module network algorithms in an automated way. This is a strong argument for using data simulators such as SynTReN for the purpose of developing, testing and improving such algorithms.

## Conclusion

We developed a module network learning algorithm called LeMoNe and tested its performance on simulated expression data sets generated by SynTReN [20]. We found that the Bayesian score can be used to infer the optimal number of modules, and that the inference performance increases as a function of the number of simulated experiments but saturates well below 1.

We also used SynTReN data to assess the effects of the methodological changes we made in LeMoNe with respect to the original methods used in Genomica [6]. Overall, application of Genomica and LeMoNe to various simulated data sets gave comparable results, with a bias towards higher recall for Genomica and higher precision for LeMoNe. However, LeMoNe offers some advantages over the original framework of Segal *et al*. [6], one of them being that the learning process is considerably faster. Another advantage of LeMoNe is the fact that the algorithm 'lets the data decide' when learning the regulatory tree structure. The partitioning of expression data inside a module is not dependent on the expression profiles of the potential regulators, but only on the module data itself. As a consequence, the assignment of 'bad' regulators (in terms of assignment entropy) to 'good' module splits (in terms of Bayesian score) might suggest missing or hidden regulators. This situation might occur if the true regulator is missing from the list of potential regulators, or if the expression of the targets cannot be related directly to the expression of the regulator, e.g., due to posttranslational regulation of the regulator's activity. We have also shown that filtering the module network by the location of regulators in the regulation program or by introducing an entropy cut-off improves the inference performance. When inferring regulatory programs from real data, these criteria may prove useful to prioritize regulators for experimental validation.

Finally, we explored the extent to which module networks inferred from real expression data could be validated using structured biological information. For that purpose, we learned module networks from a microarray compendium of stress experiments on budding yeast [21]. We found that the resulting regulatory programs overlapped only marginally with the available ChIP-chip data and GO information. However, more detailed manual analysis uncovered that the learned regulation programs are nevertheless biologically relevant, suggesting that an automated assessment of the performance of module network algorithms using structured biological information such as ChIP-chip data or GO is ineffective. This underscores the importance of using data simulators such as SynTReN for the purpose of testing and improving module network learning algorithms.

## Authors' contributions

T.M. and S.M. designed the study, developed software, analyzed the data and wrote the paper. E.B. and A.J. designed the study, developed software and analyzed the data. Y.S. designed the study and developed software. T.V.d.B. and K.V.L. developed software. P.v.R., M.K., K.M. and Y.V.d.P. designed the study and supervised the project.

## Supplementary Material

Additional file 1 Contains 2 additional figures for the precision and *F*-measure as a function of the number of modules and experiments, as well as more details about the Bayesian score and about the algorithm for learning module regulation programs. Click here for file

## References

[B1] Ideker T, Galitski T, Hood L (2001). A new approach to decoding life: systems biology. Annu Rev Genomics Hum Genet.

[B2] Ashburner M, Ball CA, Blake JA, Botstein D, Butler H, Cherry JM, Davis AP, Dolinski K, Dwight SS, Eppig JT, Harris MA, Hill DP, Issel-Tarver L, Kasarskis A, Lewis S, Matese JC, Richardson JE, Ringwald M, Rubin GM, Sherlock G (2000). Gene ontology: tool for the unification of biology. The Gene Ontology Consortium. Nat Genet.

[B3] Friedman N, Linial M, Nachman I, Pe'er D (2000). Using Bayesian networks to analyze expression data. J Comput Biol.

[B4] Pe'er D, Regev A, Elidan G, Friedman N (2001). Inferring subnetworks from perturbed expression profiles. Bioinformatics.

[B5] Bar-Joseph Z, Gerber GK, Lee TI, Rinaldi NJ, Yoo JY, Robert F, Gordon DB, Fraenkel E, Jaakkola TS, Young RA, Gifford DK (2003). Computational discovery of gene modules and regulatory networks. Nat Biotechnol.

[B6] Segal E, Shapira M, Regev A, Pe'er D, Botstein D, Koller D, Friedman N (2003). Module networks: identifying regulatory modules and their condition-specific regulators from gene expression data. Nat Genet.

[B7] Beer MA, Tavazoie S (2004). Predicting gene expression from sequence. Cell.

[B8] Friedman N (2004). Inferring cellular networks using probabilistic graphical models. Science.

[B9] Harbison CT, Gordon DB, Lee TI, Rinaldi NJ, Macisaac KD, Danford TW, Hannett NM, Tagne JB, Reynolds DB, Yoo J, Jennings EG, Zeitlinger J, Pokholok DK, Kellis M, Rolfe PA, Takusagawa KT, Lander ES, Gifford DK, Fraenkel E, Young RA (2004). Transcriptional regulatory code of a eukaryotic genome. Nature.

[B10] Luscombe NM, Madan Babu M, Yu H, Snyder M, Teichmann SA, Gerstein M (2004). Genomic analysis of regulatory network dynamics reveals large topological changes. Nature.

[B11] Xu X, Wang L, Ding D (2004). Learning module networks from genome-wide location and expression data. FEBS Lett.

[B12] Battle A, Segal E, Koller D (2005). Probabilistic discovery of overlapping cellular processes and their regulation. J Comput Biol.

[B13] Basso K, Margolin AA, Stolovitzky G, Klein U, Dalla-Favera R, Califano A (2005). Reverse engineering of regulatory networks in human B cells. Nat Genet.

[B14] Garten Y, Kaplan S, Pilpel Y (2005). Extraction of transcription regulatory signals from genome-wide DNA-protein interaction data. Nucleic Acids Res.

[B15] Petti AA, Church GM (2005). A network of transcriptionally coordinated functional modules in *Saccharomyces cerevisiae*. Genome Res.

[B16] Lemmens K, Dhollander T, De Bie T, Monsieurs P, Engelen K, Smets B, Winderickx J, De Moor B, Marchal K (2006). Inferring transcriptional modules from ChIP-chip, motif and microarray data. Genome Biol.

[B17] Van den Bulcke T, Lemmens K, Van de Peer Y, Marchal K (2006). Inferring transcriptional networks by mining 'omics' data. Current Bioinformatics.

[B18] Hartwell LH, Hopfield JJ, Leibler S, Murray AW (1999). From molecular to modular cell biology. Nature.

[B19] Segal E, Friedman N, Kaminski N, Regev A, Koller D (2005). From signatures to models: understanding cancer using microarrays. Nat Genet.

[B20] Van den Bulcke T, Van Leemput K, Naudts B, van Remortel P, Ma H, Verschoren A, De Moor B, Marchal K (2006). SynTReN: a generator of synthetic gene expression data for design and analysis of structure learning algorithms. BMC Bioinformatics.

[B21] Gasch AP, Spellman PT, Kao CM, Carmel-Harel O, Eisen MB, Storz G, Botstein D, Brown PO (2000). Genomic expression programs in the response of yeast cells to environmental changes. Mol Biol Cell.

[B22] Butte AJ, Kohane IS (2000). Mutual information relevance networks: functional genomic clustering using pairwise entropy measurements. Pac Symp Biocomput.

[B23] Butte A, Tamayo P, Slonim D, Golub T, Kohane I (2000). Discovering functional relationships between RNA expression and chemotherapeutic susceptibility using relevance networks. PNAS.

[B24] Pe'er D, Regev A, A T (2002). Minreg: Inferring an active regulator set. Bioinformatics.

[B25] Sinkkonen J, Kaski S (2002). Clustering based on conditional distributions in an auxiliary space. Neural Comput.

[B26] Kasturi J, Acharya R, Ramanathan M (2003). An information theoretic approach for analyzing temporal patterns of gene expression. Bioinformatics.

[B27] Genomica. http://genomica.weizmann.ac.il.

[B28] *Saccharomyces *Genome Database. http://www.yeastgenome.org/.

[B29] Ma HW, Kumar B, Ditges U, Gunzer F, Buer J, Zeng AP (2004). An extended transcriptional regulatory network of *Escherichia coli *and analysis of its hierarchical structure and network motifs. Nucleic Acids Res.

[B30] Segal E, Pe'er D, Regev A, Koller D, Friedman N (2005). Learning module networks. Journal of Machine Learning Research.

[B31] Heller KA, Ghahramani Z (2005). Bayesian hierarchical clustering. Proceedings of the twenty-second International Conference on Machine Learning.

[B32] Shannon CE (1948). A mathematical theory of communication. The Bell System Technical Journal.

[B33] de Hoon MJL, Imoto S, Nolan J, Miyano S (2004). Open source clustering software. Bioinformatics.

[B34] Maere S, Heymans K, Kuiper M (2005). BiNGO: a Cytoscape plugin to assess overrepresentation of gene ontology categories in biological networks. Bioinformatics.

[B35] SynTReN. http://homes.esat.kuleuven.be/~kmarchal/SynTReN.

[B36] LeMoNe. http://bioinformatics.psb.ugent.be/LeMoNe/download.htm.

