# Supplementary material for: Validating module network learning algorithms using simulated data
Source: BMC Bioinformatics. 2007 May 3;8(Suppl 2):S5. doi: 10.1186/1471-2105-8-S2-S5 (PMC1892074; doi:10.1186/1471-2105-8-S2-S5)
Supplement: Additional file 1 — Contains 2 additional figures for the precision and F-measure as a function of the number of modules and experiments, as well as more details about the Bayesian score and about the algorithm for learning module regulation programs. [file 1471-2105-8-S2-S5-S1.pdf]

Validating module network learning algorithms using simulated data: Supplementary information

Precision and F-measure as a function of the number of modules and experiments

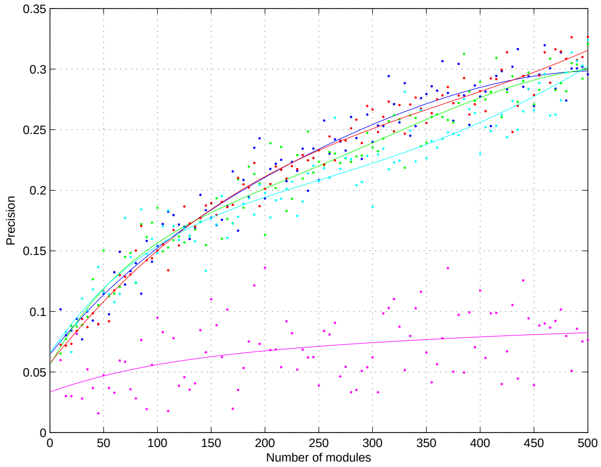

Figure S1: Precision as a function of the number of modules for data sets with 10 (magenta), 50 (cyan), 100 (red), 200 (green), and 300 (blue) experiments. The curves are least squares fits of the data to a linear non-polynomial model of the form $a_0+\sum_{k=1}^n a_k x^{k-1} e^{-x/500}$ with x the number of modules and $n=3$.

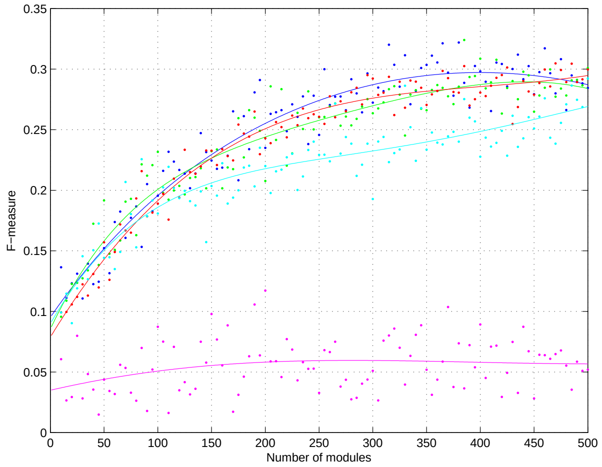

Figure S2: F-measure as a function of the number of modules for data sets with 10 (magenta), 50 (cyan), 100 (red), 200 (green), and 300 (blue) experiments. The curves are least squares fits of the data to a linear non-polynomial model of the form $a_0+\sum_{k=1}^n a_k x^{k-1} e^{-x/500}$ with x the number of modules and $n=3$.

Module networks Bayesian score

We use the same Bayesian score as in the original module networks formalism [1, 2]. The data likelihood is given by evaluating the module network joint probability distribution (eq. (2)) on the data set, assuming independent experiments,

$$\mathcal{L} = \prod_{m=1}^{M} \prod_{k=1}^{K} \prod_{i\in\mathcal{A}_k} p_k\big(x_{i,m} \mid \{x_{j,m} \colon j\in\Pi_k\}\big),$$

where $x_{i,m}$ is the log-normalized expression value of gene i in experiment m.

The Bayesian score is obtained by taking the log of the marginal probability of the data likelihood over the parameters of the normal distributions at the leaves of the regression trees with a normal-gamma prior. It decomposes as a sum of leaf scores of the different modules:

$$\mathbf{S} = \sum_k \mathbf{S}_k = \sum_k \sum_\ell S_k(\mathcal{E}_\ell)$$
$$S_k(\mathcal{E}_\ell) = \log \iint d\mu d\tau\, p(\mu,\tau) \prod_{m\in\mathcal{E}_\ell} \prod_{i\in\mathcal{A}_k} p_{\mu,\tau}(x_{i,m}), \tag{S1}$$

where k runs over the set of modules and ℓ runs over the set of leaves of the regression tree of module k; $\mathcal{E}_\ell$ denotes the experiments that end up at leaf ℓ after traversing the regression tree and $\mathcal{A}_k$ denotes the genes assigned to module k; $p(\mu,\tau)$ is a normal-gamma distribution over the mean μ and precision τ of the normal distribution $p_{\mu,\tau}$, i.e., $p(\mu,\tau) = p(\mu \mid \tau)p(\tau)$ where $p(\tau) \sim \Gamma(\alpha_0,\beta_0)$ and $p(\mu \mid \tau) \sim \mathcal{N}(\mu_0,(\lambda_0\tau)^{-1})$:

$$p(\tau) = \frac{\beta_0^{\alpha_0}}{\Gamma(\alpha_0)} \tau^{\alpha_0 - 1} e^{-\beta_0 \tau} \quad \text{(S2)}$$

$$p(\mu \mid \tau) = \big(\frac{\lambda_0 \tau}{2\pi}\big)^{1/2} e^{-\frac{\lambda_0 \tau}{2}(\mu - \mu_0)^2} \quad \text{(S3)}$$

$$p_{\mu,\tau}(x_{i,m}) = \big(\frac{\tau}{2\pi}\big)^{1/2} e^{-\frac{\tau}{2}(x_{i,m} - \mu)^2} \quad \text{(S4)}$$

with $\alpha_0, \beta_0, \lambda_0 > 0$ and $-\infty < \mu_0 < \infty$.

Insertion of eqs. (S2)–(S4) into eq. (S1) leads to an integral that can be solved explicitly as a function of the sufficient statistics

$$R_q^{(\ell)} = \sum_{m \in \mathcal{E}_\ell} \sum_{i \in \mathcal{A}_k} x_{i,m}^q \ , \ q = 0, 1, 2.$$

of the leaves of the regression tree. The result is

$$\begin{aligned} S_k(\mathcal{E}_\ell) = &- \tfrac{1}{2} R_0^{(\ell)} \log(2\pi) + \tfrac{1}{2} \log\big(\frac{\lambda_0}{\lambda_0 + R_0^{(\ell)}}\big) \\ &- \log\Gamma(\alpha_0) + \log\Gamma(\alpha_0 + \tfrac{1}{2} R_0^{(\ell)}) \\ &+ \alpha_0 \log\beta_0 - (\alpha_0 + \tfrac{1}{2} R_0^{(\ell)}) \log\beta_1 \quad \text{(S5)} \end{aligned}$$

where

$$\beta_1 = \beta_0 + \frac{1}{2} \Big[R_2^{(\ell)} - \frac{(R_1^{(\ell)})^2}{R_0^{(\ell)}} \Big] + \frac{\lambda_0 \big(R_1^{(\ell)} - \mu_0 R_0^{(\ell)}\big)^2}{2(\lambda_0 + R_0^{(\ell)}) R_0^{(\ell)}}.$$

Learning module regulation programs

The pseudocode for the regulation program learning algorithm is given in Figure S3. In its simplest form, the merge score for two trees T_{α_1} and T_{α_2} considers only the gain in Bayesian score that is obtained by merging two sets into one:

$$r_{\alpha_1,\alpha_2} = S_k(\mathcal{E}_{\alpha_1} \cup \mathcal{E}_{\alpha_2}) - S_k(\mathcal{E}_{\alpha_1}) - S_k(\mathcal{E}_{\alpha_2}). \quad \text{(S6)}$$

In our computations we used a merge score which is slightly more complicated and takes into account the whole substructure of the tree below T_{α_1} and T_{α_2}.

Let T_α be a tree with children T_{α_1} and T_{α_2}, and define recursively

$$Z_\alpha = e^{S_k(\mathcal{E}_\alpha)} + Z_{\alpha_1} Z_{\alpha_2}$$

with initial condition

$$Z_m = e^{S_k(\{m\})}$$

for the trivial tree with one experiment m and no children. The new merge score is then defined as

$$r_{\alpha_1,\alpha_2} = S_k(\mathcal{E}_{\alpha_1} \cup \mathcal{E}_{\alpha_2}) - \ln Z_{\alpha_1} - \ln Z_{\alpha_2}. \quad \text{(S7)}$$

A binary tree T_α generates a nested set of partitions $\mathcal{P}_\alpha$ (we write this as $\mathcal{P}_\alpha \sim T_\alpha$) of its experiment set $\mathcal{E}_\alpha$ and to each such partition corresponds a score

$$\mathbf{S}_k(\mathcal{P}_\alpha) = \sum_i S_k(\mathcal{E}_i)$$

where $\mathcal{E}_i$ are the subsets of $\mathcal{E}_\alpha$ forming the partition $\mathcal{P}_\alpha$. Since a partition generated by T_α is either the singleton partition $\mathcal{P}_\alpha = \{\mathcal{E}_\alpha\}$, or a combination of a partition generated by T_{α_1} with a partition generated by T_{α_2}, we get immediately

$$Z_\alpha = \sum_{\mathcal{P}_\alpha \sim T_\alpha} e^{\mathbf{S}_k(\mathcal{P}_\alpha)},$$

or

$$\ln Z_\alpha = S_k(\mathcal{E}_\alpha) + \ln\Big(1 + \sum_{\substack{\mathcal{P}_\alpha \sim T_\alpha \\ \mathcal{P}_\alpha \neq \{\mathcal{E}_\alpha\}}} e^{\mathbf{S}_k(\mathcal{P}_\alpha) - S_k(\mathcal{E}_\alpha)}\Big) \quad \text{(S8)}$$

We conclude that the merge score (S7) contains the score difference (S6) as well as other terms defined by the structure of the subtrees T_{α_1} and T_{α_2}. If two pairs of trees give the same score difference (S6), the merge score (S7) will typically favor to merge the pair with the smallest substructure first (as the number of terms in the summation in (S8) is smaller). Hence, using (S7) instead of (S6) leads to more balanced trees.

Since we are building the tree from the bottom up, computing the partition sums Z_α is done along the way, and using the merge score (S7) instead of (S6) comes at no computational cost. The whole process of constructing the tree with a merge score depending on all the partitions generated by the subtrees is very similar to the Bayesian hierarchical clustering method of [3].

Regulator assignment in the presence of missing values

In real data there are often missing values, so for some experiments we do not know if a regulator is above or below a given split value. Using the non-missing values to define the sets $\mathcal{R}_1$ and $\mathcal{R}_2$, we

compute q_i as before. Since regulators with a lot of missing values lead to more uncertainty, we penalize those by moving the conditional probabilities q_i closer to the maximum uncertainty value of $\frac{1}{2}$ by defining

$$q_i' = (1 - \tfrac{|\mathcal{R}_3|}{|\mathcal{E}_\alpha|})\,(q_i - \tfrac{1}{2}) + \tfrac{1}{2},$$

where

$$\mathcal{R}_3 = \{m \in \mathcal{E}_\alpha \colon x_{r,m} \text{ is missing}\}.$$

Note that when there are no missing values, $q_i' = q_i$, and in the extreme case where there are only missing values, $q_i' = \frac{1}{2}$. For the probability distribution of R, we distribute the missing values proportionally over 1 and 2,

$$p_i' = \frac{|\mathcal{R}_i| + \frac{|\mathcal{R}_i|}{|\mathcal{R}_1|+|\mathcal{R}_2|}|\mathcal{R}_3|}{|\mathcal{E}_\alpha|}.$$

such that $p_1'+p_2'$ still sums up to 1. We now minimize the conditional entropy

$$H(E \mid R) = p_1' h(q_1') + p_2' h(q_2')$$

corresponding to these modified probability distributions. For the sufficient statistics of the leaves of the module, we simply ignore the missing values as there are typically more than enough combined data points for a reliable computation of those statistics.

The complete regulator assignment algorithm is given in Supplementary Figure S4

```
/* Find hierarchical tree                        */
Input: A list treeList of trivial trees
       representing single experiments.
while treeList has more than 1 element do
    compute r_{α1,α2} for each pair of trees in
    treeList;
    construct the joined tree T_α = T_{α1} ∪ T_{α2}
    for the pair with highest r_{α1,α2};
    add T_α to treeList and remove T_{α1}, T_{α2};
Output: A single tree T_0 representing all
        experiments.

/* Find optimal regulation program
   leaves                                        */
testScore(T_0.root);

/* Recursive procedure to cut the
   hierarchical tree                             */
Begin testScore(node)
    if S_k(node) < S_k(node.leftChild) +
    S_k(node.rightChild) then
        testScore(node.leftChild);
        testScore(node.rightChild);
    else
        cut tree below node;
End
```

Figure S3: Pseudocode for the regulation program learning method

```
/* Assign regulators separately for
   different regulation tree levels.
   */
/* Level is the distance from a node
   to the root.                              */
for each level l do
    create a list nodeList with all nodes at
    level l in the trees of all modules;
    while nodeList is not empty do
        for each node in nodeList do
            for each regulator r in the set of
            potential regulators that do not
            break acyclicity do
                compute the entropy for
                assigning r to node;
        find the node-regulator pair
        (bestNode, bestR) with least
        entropy;
        assign bestR to bestNode;
        remove bestNode from nodeList;
```

Figure S4: Pseudocode for the regulation program learning method

References

1. Segal E, Shapira M, Regev A, Pe'er D, Botstein D, Koller D, Friedman N: **Module networks: identifying regulatory modules and their condition-specific regulators from gene expression data**. *Nat Genet* 2003, **34**:166 – 167.

2. Segal E, Pe'er D, Regev A, Koller D, Friedman N: **Learning module networks**. *Journal of Machine Learning Research* 2005, **6**:557 – 588.

3. Heller KA, Ghahramani Z: **Bayesian hierarchical clustering**. In *Proceedings of the twenty-second International Conference on Machine Learning* 2005.
